# Maternal Glycemic Spectrum and Adverse Pregnancy and Perinatal Outcomes in a Multiracial US Cohort

**DOI:** 10.3390/jcdd9060179

**Published:** 2022-06-04

**Authors:** Yaa Adoma Kwapong, Ellen Boakye, Guoying Wang, Xiumei Hong, Jennifer Lewey, Mamas Andreas Mamas, Pensee Wu, Michael Joseph Blaha, Khurram Nasir, Allison Gamboa Hays, Roger Scott Blumenthal, Xiaobin Wang, Garima Sharma

**Affiliations:** 1 Johns Hopkins Ciccarone Center for the Prevention of Cardiovascular Diseases, Johns Hopkins School of Medicine, 600 N Wolfe St, Baltimore, MD 21287, USA; eboakye2@jhmi.edu (E.B.); mblaha1@jhmi.edu (M.J.B.); knasir1@jhmi.edu (K.N.); ahays2@jhmi.edu (A.G.H.); rblument@jhmi.edu (R.S.B.); gsharma8@jhmi.edu (G.S.); 2 Department of Population, Family and Reproductive Health, Johns Hopkins Bloomberg School of Public Health, 615 N Wolfe St, Baltimore, MD 21205, USA; gwang24@jhu.edu (G.W.); xhong3@jhu.edu (X.H.); xwang82@jhu.edu (X.W.); 3 Division of Cardiology, Hospital of University of Pennsylvania, Philadelphia, PA 19104, USA; jennifer.lewey@pennmedicine.upenn.edu; 4 Keele Cardiovascular Research Group, School of Medicine, Keele University, Keele ST5 5BG, UK; mamasmamas1@yahoo.co.uk; 5 Division of Maternal and Fetal Medicine, Keele University, Keele ST5 5BG, UK; p.wu@keele.ac.uk; 6 Center for Outcomes Research, Houston Methodist Hospital and DeBakey Heart & Vascular Center, Houston, TX 77030, USA; 7 Division of Cardiovascular Prevention and Wellness, Department of Cardiology, Houston Methodist Debakey Heart and Vascular Center, Houston, TX 77030, USA; 8 Department of Pediatrics, Johns Hopkins University School of Medicine, Baltimore, MD 21287, USA

**Keywords:** glycemia, gestational diabetes, adverse pregnancy outcomes, cardiovascular risk

## Abstract

Diabetes mellitus (pregestational (PDM) and gestational (GDM)) is associated with adverse pregnancy outcomes (APOs). However, studies exploring the association of APOs with maternal glycemia among women without PDM/GDM are limited. We utilized data from 4119 women (307—PDM; 582—GDM; 3230—non-PDM/GDM) in the Boston Birth Cohort (1998–2016). Women in the non-PDM/GDM group were subdivided by tertiles of 1 h, 50 g oral glucose load test at 24–32 weeks: T1: 50–95 mg/dL (*n* = 1166), T2: 96–116 mg/dL (*n* = 1151), T3: 117–201 mg/dL (*n* = 913). Using multivariable logistic regression, we examined the association of maternal glycemia with APOs—preterm birth (PTB) and hypertensive disorders of pregnancy (HDP)—and adverse perinatal outcomes—high birth weight (HBW), cesarean section (CS), and sub-analyses by race-ethnicity. Compared to women in T1, women in T2 and T3 had a higher prevalence of pre-existing hypertension (T1: 2.8% vs. T2: 5.2% vs. T3: 6.3%) and obesity (T1: 13.3% vs. T2: 18.1% vs. T3: 22.9%). Women in T2 and T3 had higher odds of HBW (adjusted odds ratio aOR T2: 1.47 [1.01–2.19] T3: 1.68 [1.13–2.50]) compared to women in T1. Additionally, women in T2, compared to T1, had higher odds of HDP (aOR 1.44 [1.10–1.88]). Among non-Hispanic Black (NHB) women, those in T2 and T3 had higher odds of HDP compared to T1 (aOR T2 1.67 [1.13–2.51]; T3: 1.68 [1.07–2.62]). GDM and PDM were associated with higher odds of HBW, CS, PTB, and HDP, compared to women in T1. In this predominantly NHB and Hispanic cohort, moderate maternal glycemia without PDM/GDM was associated with higher odds of HBW and HDP, even more strongly among NHB women. If confirmed, a review of current guidelines of glucose screening and risk stratification in pregnancy may be warranted.

## 1. Introduction

Adverse pregnancy outcomes (APOs), which include hypertensive disorders of pregnancy (HDP), preterm birth (PTB), and intrauterine growth restriction, affect approximately 1 in 5 pregnancies in the US [1]. Mechanisms underlying this group of interrelated disorders include placental dysfunction, maternal vascular endothelial dysfunction, inflammation, and vasospasm [2,3,4]. APOs such as preeclampsia and PTB are among the leading causes of maternal and neonatal morbidity and mortality [5,6,7,8], and they are associated with increased short- and long-term cardiovascular risk [4,9,10,11] and long-term kidney disease in women [12]. A history of multiple APOs elevates the risk of cardiovascular disease even further [11]. Thus, the burden of APOs on both mother and fetus cannot be overemphasized.

Diabetes complicates approximately 16.8% of all pregnancies: 13.6% pregestational (PDM; diagnosed before pregnancy) and 86.4% gestational diabetes (GDM; defined as any degree of glucose intolerance with onset or first recognition during pregnancy), according to the International Diabetes Federation [13]. In 2016, the prevalence of PDM and GDM in the US were estimated to be 0.9% and 6.0%, respectively [14]. GDM is associated with other APOs such as PTB [15,16], preeclampsia [16], perinatal outcomes such as macrosomia [15,16,17,18,19], increased rate of cesarean section [15,16,19], and neonatal hypoglycemia [15]. In addition, women with a history of GDM have an increased risk of future cardiovascular disease [1,20], and increased rates of type 2 diabetes and obesity in their offspring [21]. Therefore, diagnosis of GDM is aimed primarily at identifying women at risk of short- and long-term adverse outcomes.

Although there is substantial evidence that PDM and GDM are associated with APOs and adverse perinatal outcomes, studies exploring various degrees of maternal glycemia outside the range of PDM or GDM with these outcomes are limited. This study examines the association of APOs and perinatal outcomes with maternal glycemic status across the entire spectrum, including normal glycemic gradient, and sub-analyses by race-ethnicity. It also describes the differential prevalence of key traditional cardiovascular risk factors between these maternal glycemic subgroups in an urban, predominantly underrepresented U.S. population.

## 2. Methods

### 2.1. Data Source, Study Design, and Study Population

We utilized data from the Boston Birth Cohort (BBC; 1998–2016) in this cross-sectional study. The BBC is a predominantly urban, low-income, ethnic minority population of mother–baby dyads recruited from the Boston Medical Center (1998–2016). It was originally designed as a case-control study to explore the genetic and environmental determinants of preterm birth and low birth weight [22]. Eligibility criteria into the BBC was the delivery of a singleton birth at the Boston Medical center. Eligible mothers who agreed to participate were recruited within 24 to 72 h after providing written informed consent. Pregnancies resulting from in vitro fertilization, multiple pregnancies, and newborns with major birth defects were excluded. Data on maternal sociodemographic characteristics were collected via a standardized questionnaire. Pregnancy, perinatal outcomes, and cardiovascular risk factors were abstracted from the electronic medical records. A detailed description of the BBC has been previously described [23].

The BBC study protocol was approved by the Institutional Review Boards of Boston University Medical Center, and the Johns Hopkins Bloomberg School of Public Health. This current study is within the scope of the IRB approval. We followed the Strengthening the Reporting of Observational Studies in Epidemiology guidelines. This current study utilized data from 4119 women who had data available on glycemic status as well as adverse pregnancy and perinatal outcomes.

### 2.2. Assessment of Primary Outcomes

The primary outcomes were adverse pregnancy outcomes which included PTB and HDP. PTB was defined as delivery < 37 weeks of gestation per the guidelines of the American College of Obstetricians and Gynecologists [24]. Early prenatal ultrasonography was used to assess gestational age. In the absence of that, an algorithm using the first day of the last menstrual period was used [23]. HDP included gestational hypertension, preeclampsia, eclampsia, and hemolysis; elevated liver enzymes; and low platelets (HELLP) syndrome. These were based on physician diagnoses and extracted from medical records. Preeclampsia was defined as systolic blood pressure ≥ 140 mmHg or diastolic blood pressure ≥ 90 mmHg, and proteinuria ≥ 1+ on at least two occasions with onset after 20 weeks of gestation or worsening chronic hypertension (systolic blood pressure ≥ 160 mmHg or diastolic blood pressure ≥ 110 mmHg), based on the National High Blood Pressure Education Program Working Group on High Blood Pressure in Pregnancy [25].

### 2.3. Assessment of Secondary Outcomes

Secondary outcomes included perinatal outcomes such as birth weight and cesarean section, abstracted from the electronic medical records. Birth weight was modeled as a continuous variable and as a binary variable—high birth weight (HBW yes or no). HBW was defined as birth weight ≥ 4000 g.

### 2.4. Glycemic Status

Maternal blood glucose screening was performed at 24–32 weeks gestation using the 1 h, 50 g oral glucose load screening test. A glucose load of 50 g was given, and a blood draw was taken after 1 h for glucose analysis, using routine venipuncture protocol. Blood samples were analyzed in the Boston Medical Centre Laboratories using standard enzymatic methods (hexokinase) on an automated platform by Abbott Laboratories, Inc. (Salt Lake City, UT, USA) [22].

Glycemic status was classified into the following subgroups: PDM, GDM, and non-PDM/GDM. PDM and GDM were based on physician diagnoses and extracted from the electronic medical records. GDM diagnosis at the time was via the two-step approach per the guidelines of American Diabetes Association and American College of Obstetricians and Gynecologists [26,27]. This involved the initial glucose challenge test of a non-fasting 50 g glucose load, followed by a 1 h plasma glucose measurement. Women exceeding a recommended threshold proceeded to the confirmatory 100 g oral glucose tolerance test (OGTT). American College of Obstetricians and Gynecologists recommends any of the commonly used thresholds: 130 mg/dL, 135 mg/dL, or 140 mg/dL [26,27]. A threshold of 140 mg/dL was used. Women without a diagnosis of PDM or GDM were classified as the non-PDM/GDM group. Based on their 1 h, 50 g glucose load test results, they were subdivided into tertiles: T1: 50–95 mg/dL, T2: 96–116 mg/dL, T3: 117–201 mg/dL. The confirmatory 3 h, 100 g OGTT results of women in T3 with 1 h glucose ≥ 140 mg/dL did not meet the criteria for GDM.

### 2.5. Assessment of Covariates

Covariates included self-reported maternal characteristics such as maternal age in years (<20, 20–34, >35), parity (0, ≥1), race-ethnicity (Non-Hispanic Black (NHB), Hispanic, and others), and educational status (secondary/less, general education development GED/high school, and college and above). In addition, key cardiovascular risk factors assessed were pre-existing hypertension (no, yes), which was based on physician diagnoses abstracted from electronic medical records for the index pregnancy, self-reported smoking in index pregnancy (no, yes), and pre-pregnancy body mass index (BMI < 25 kg/m^2^, ≥25 kg/m^2^), which was calculated from self-reported pre-pregnancy height and weight. Infant characteristics such as sex, gestational age at delivery, and birth weight were abstracted from the medical records.

### 2.6. Statistical Analysis

Maternal sociodemographic and cardiovascular risk factors and neonatal characteristics were summarized using proportions, and differences were assessed using the chi-squared test. Multivariable logistic regression models were used to assess the association of maternal glycemic status and APO, except for birth weight, which was assessed with multivariable linear regression. For each outcome, model 1 was unadjusted, and model 2 was adjusted for potential confounders including maternal age, race, parity, educational status, smoking in index pregnancy, pre-pregnancy BMI, sex of infant, birth weight (except for outcomes birth weight and HBW), and preterm birth (except for outcomes PTB and HDP). Sub-analyses by race-ethnicity were performed. As spontaneous PTB differs from medically indicated PTB, a sensitivity analysis was performed, restricting our outcome to spontaneous PTB. The APOs and perinatal outcomes were combined as a cumulative APO score (0, 1, 2, 3+) Using ordinal logistic regression, we further explored the outcome of cumulative APO score with maternal glycemic spectrum, unadjusted, and adjusted for maternal age, race, parity, smoking, and body mass index. All statistical analyses were conducted using Stata IC version 16, and a two-sided alpha (α) of <0.05 was used to determine statistical significance of the results.

## 3. Results

Of the 4119 women, 307 had PDM, 582 had GDM, and 3230 were non-PDM/GDM (T1: 50–95 mg/dL, *n* = 1166; T2: 96–116 mg/dL, *n* = 1151; T3: 117–201 mg/dL, *n* = 913).

### 3.1. Sociodemographic and Cardiovascular Risk Factors by Maternal Glycemic Subgroups

Compared to the other maternal glycemic subgroups, women with PDM had the highest proportion of older women (age ≥ 35 years PDM: 30% vs. GDM: 29.7% vs. T3: 22.8% vs. T2: 14.9% vs. T1: 11.4%, *p* < 0.001), obesity (BMI ≥ 30; PDM: 39.4% vs. GDM: 36.4% vs. T3: 22.9% vs. T2: 18.1% and T1: 13.3%, *p* < 0.001), and smoking during the index pregnancy (PDM: 15.3% vs. GDM: 9.5% vs. T3: 11.4 vs. T2: 11.1 vs. T1: 14.8, *p* = 0.004) (Table 1). Women with PDM had the highest prevalence of pre-existing hypertension (PDM: 23.8% vs. GDM: 9.6% vs. T3: 6.3% vs. T2: 5.2% vs. T1: 2.8%, *p* < 0.001) and PTB (PDM: 43.0% vs. GDM: 33.0% vs. T3: 20.7% vs. T2: 20.8% vs. T1: 20.2%, *p* < 0.001) compared to the other glycemic groups, whilst women with GDM had the highest prevalence of HBW (BW ≥ 4000 g GDM: 14.1%, PDM: 7.2% T3: 7.4%. T2: 6.2%, T1: 4.9%, *p* < 0.001).

Among the non-PDM/GDM group, compared to T1, women in T2 and T3 had a higher prevalence of cardiovascular risk factors such as obesity (T1: 13.3% vs. T2: 18.1 vs. T3: 22.9%, *p* < 0.001) and pre-existing hypertension (T1: 2.8% vs. T2: 5.2% vs. T3: 6.3%, *p* < 0.001).

### 3.2. Maternal Glycemia and Adverse Pregnancy Outcomes

#### 3.2.1. Hypertensive Disorders in Pregnancy

**Figure 1:**
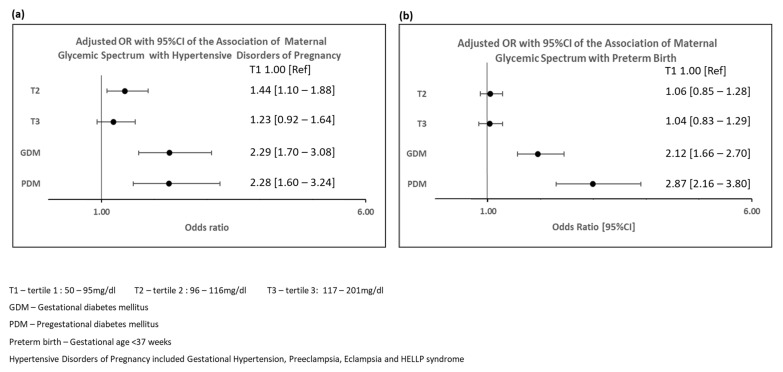
Adjusted Odds Ratios and 95% Confidence Intervals Showing Association of Maternal Glycemic Spectrum with Hypertensive Disorders of Pregnancy and Preterm Birth. (**a**) Adjusted Odds Ratios and 95% Confidence Intervals Showing Association of Maternal Glycemic Spectrum with Hypertensive Disorders of Pregnancy; (**b**). Adjusted Odds Ratios and 95% Confidence Intervals Showing Association of Maternal Glycemic Spectrum with Preterm Birth.

Compared to women in T1, women with GDM and PDM had higher odds of HDP following adjustment for differences in baseline covariates (GDM: aOR: 2.29, 95%CI: 1.70–3.08, PDM: aOR: 2.28, 95%CI: 1.60–3.24) (Figure 1a)**.** Women in the non-PDM/GDM subgroup T2 had higher odds of HDP compared to T1 (aOR: 1.44, 95%CI: 1.10–1.88).

When the non-PDM/GDM group were sub-stratified by the presence of pre-existing hypertension or not, women in the higher glucose tertiles with no pre-existing hypertension had higher odds of HDP compared to women in T1 with no pre-existing hypertension. (T2 with no pre-existing hypertension: aOR: 1.47; 95%CI: 1.11–1.93, T3 with no pre-existing hypertension: aOR: 1.37; 95%CI: 1.03–1.82) (Table 2). Additionally, the association of maternal glycemia with HDP was much stronger in women with pre-existing hypertension, compared to women in T1 with no pre-existing hypertension (T1 with pre-existing hypertension: aOR: 7.63; 95%CI: 3.81–15.3, T2 with pre-existing hypertension: aOR: 5.91; 95%CI: 3.36–10.40, T3 with pre-existing hypertension: aOR: 6.59; 95%CI: 3.89–11.17) (Table 2).

#### 3.2.2. Preterm Birth

Women with GDM and PDM had higher odds of PTB compared to T1 (GDM: aOR 2.12, 95%CI: 1.66–2.70, PDM: aOR: 2.87, 95%CI: 2.16–3.80) (Supplementary Table S1 and Figure 1b).

However, the odds of PTB in T2 and T3 were not significantly different from T1 (T2: aOR: 1.06, 95%CI: 0.86–1.30; T3: aOR: 1.04, 95%CI: 0.83–1.29). When outcome was restricted to spontaneous PTB, the results were consistent (Supplementary Table S2).

### 3.3. Maternal Glycemia and Perinatal Outcomes

#### 3.3.1. Birth Weight

Women with GDM and PDM had higher odds of HBW compared to T1 after adjusting for confounders (GDM: aOR: 3.62, 95%CI: 2.42–5.41; PDM: aOR: 1.89, 95%CI: 1.09–3.28). Similarly, compared to T1, T2 and T3 were associated with higher odds of HBW (T2: aOR: 1.47, 95%CI: 1.01–2.19; T3: aOR 1.68, 95%CI 1.13–2.50) in a graded fashion (Supplementary Table S3).

#### 3.3.2. Cesarean Section

Compared to T1, GDM and PDM were significantly associated with higher odds of CS in adjusted models (GDM: aOR: 1.63, 95%CI: 1.31–2.02; PDM: aOR: 2.20, 95%CI: 1.68–2.89) (Supplementary Table S4). In contrast, T2 and T3 were not significantly associated with CS after adjusting for pertinent sociodemographic and cardiovascular risk factors (T2: aOR: 1.16, 95%CI: 0.97–1.39; T3: aOR: 1.06, 95%CI: 0.87–1.28).

### 3.4. Maternal Glycemia and Adverse Pregnancy Outcomes by Race-Ethnicity

#### 3.4.1. Hypertensive Disorders of Pregnancy

Among NHB women, those in T2 and T3, compared to T1 had higher odds of HDP after adjustment for confounders (T2: aOR: 1.67, 95%CI: 1.13–2.51; T3: aOR: 1.68, 95%CI: 1.13–2.51), in a similar pattern to GDM (OR: 2.60, 95%CI: 1.60–4.21, aOR: 2.09, 95%CI: 1.25–3.49) and PDM (OR: 2.30, 95%CI: 1.31–4.04; aOR: 1.84, 95%CI: 1.02–3.32) (Table 3). Among Hispanic women, only women with PDM had higher odds of HDP compared to T1 (aOR: 2.40, 95%CI: 1.07–5.39). Among women of other race-ethnicity groups, T2 (aOR: 1.63, 95%CI: 1.09–2.44), GDM (aOR: 2.60, 95%CI: 1.68–4.05), and PDM (aOR: 2.51, 95%CI: 1.47–4.30) were associated with higher odds of HDP compared to T1.

#### 3.4.2. Preterm Birth

Compared to T1, only GDM and PDM were significantly associated with high odds of PTB among NHB (GDM: aOR: 1.65, 95%CI: 1.07–2.55; PDM: aOR: 3.66, 95%CI: 2.31–5.80), Hispanic (GDM: aOR: 2.09, 95%CI: 1.19–3.68; PDM: 2.68, 95%CI: 1.37–5.24), and women of other race-ethnicity groups (GDM: aOR: 2.40, 95%CI: 1.69–3.43; PDM: aOR: 2.35, 95%CI: 1.52–3.64) (Supplementary Table S5). The odds of PTB in the higher tertiles of non-PDM/GDM group were not significantly different from the lowest in all the race-ethnicity groups.

### 3.5. Maternal Glycemia and Perinatal Outcomes by Race-Ethnicity

#### 3.5.1. Birth Weight

Compared to women in T1, T3 and GDM were linearly positively associated with birth weight among NHB women (T3: adjusted β: 127.88, *p* = 0.002, GDM: adjusted β: 172.47, *p* = 0.001), Hispanic women (T3: adjusted β: 175.65, *p* = 0.010, GDM: adjusted β: 218.53, *p* = 0.003), and women of other race-ethnicity groups (T3: adjusted β: 126.88, *p* < 0.001, GDM: adjusted β: 174.62, *p* < 0.001) (Supplementary Table S6). Among NHB women, T3 and GDM had higher odds of HBW compared to those in T1. (T3: aOR: 2.01, 95%CI: 1.08–3.74, GDM: aOR: 3.87, 95%CI: 1.97–7.59) (Supplementary Table S7).

#### 3.5.2. Cesarean Section

Among NHB women, GDM (aOR: 1.55, 95%CI: 1.06–2.27) and PDM (aOR: 2.12, 95%CI: 1.36–3.30) were associated with higher odds of CS compared to T1 (Supplementary Table S8). Among Hispanic women, only PDM was associated with higher odds of CS (aOR: 2.68, 95%CI: 1.40–5.15). Among women of other race-ethnicities, the odds of CS in GDM and PDM were two times higher than that of T1 (GDM: aOR: 2.32, 95%CI: 1.68–3.21; PDM: aOR: 2.14, 95%CI: 1.41–3.25. There was no significant association of T2 and T3 with CS in all the race-ethnicity groups.

**Figure 2:**
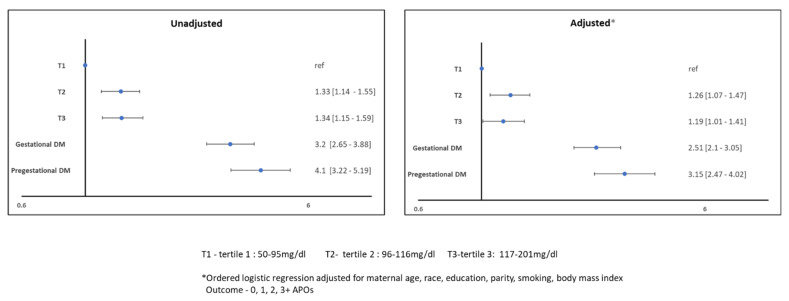
Association of Cumulative APOs with Maternal Glycemic Spectrum.

A cumulative APO score was explored with maternal glycemic spectrum and the results are shown in Figure 2.

## 4. Discussion

In this sample of racially diverse, predominantly underrepresented women, we found that both GDM and PDM were associated with higher odds of HBW, CS, PTB, and HDP. Importantly, our findings demonstrate that women with moderate glycemia (T2: 96–116 mg/dL and T3: 117–201 mg/dL) also have higher odds of HBW and HDP, particularly among non-Hispanic Black women. Furthermore, women with moderate glycemia that did not make the cut-off for GDM had a higher prevalence of traditional cardiovascular risk factors, such as obesity and chronic hypertension, compared to those in the lower tertile of glycemia.

The novel finding of our study is that moderate glycemia in non-PDM/GDM women is associated with HDP, and when stratified by race, this association is stronger among NHB women. This is an important finding with relevant clinical implications for women at elevated risks of developing HDP. Our findings of a stronger association of maternal glycemia with HDP in NHB women may inform clinicians of elevated risks in these women that need to be addressed with prenatal cardiometabolic risk assessment and healthy lifestyle initiation. Pregnancy provides a critical window of opportunity to discuss cardiovascular risk factors that have strong implications on APOs and long-term cardiovascular risk. Women with moderate glycemia that do not meet the criteria for GDM might be an additional group with an elevated risk of HDP.

Our finding that PDM, GDM, and moderate glycemia in non-PDM/GDM women were associated with birth weight is consistent with other studies [19,28]. In a systematic review and meta-analysis of 25 cohort studies across 207,172 women, positive linear associations were found between post-load glucose concentration and adverse perinatal outcomes, such as large for gestational age, shoulder dystocia, cesarean delivery, and neonatal hypoglycemia, with no clear evidence of threshold effect [19]. Similarly consistent with this finding is the Hyperglycemia and Adverse Pregnancy Outcomes (HAPO) study by Metzger et al., a large multicenter prospective US-based cohort study involving over 25,000 pregnant women, in which increasing levels of 1 h plasma glucose on OGTT were found to be associated with birth weight above 90th percentile [28]. Our study is unique as it additionally explores HDP and PTB, and further, by race-ethnicity. These findings support the over-60-year-old hypothesis formulated by Jorgen Pedersen, which attributes fetal overgrowth to the increased transplacental transfer of glucose from maternal blood to the fetus [29]. This stimulates fetal beta cells to produce insulin (fetal hyperinsulinism) and results in a subsequent increase in fetal growth due to the anabolic effects of insulin. The extended Pederson hypothesis posits that aside from the intrinsic fetal hyperinsulinism that causes the initial increase in fetal weight, limited fetal oxygen availability leads to an altered utilization of glucose by tissues, an increased fetal adipocyte alpha-glycerophosphate synthesis, and increased fetal adiposity [29]. Though this is well established at high levels of glycemia, our findings suggest that these mechanisms may exist at moderate levels of glycemia as well.

We found that GDM and PDM were associated with CS and PTB, consistent with previous research [15,16,19,30], but no significant association was found with moderate glycemia with CS and PTB. In addition, we reported that GDM and PDM were associated with HDP, comparable to findings from a large case-control study, which showed that women with GDM had a 1.5-fold increased risk in preeclampsia and 1.5-fold increased risk overall of developing a hypertensive disorder of pregnancy [31]. That same study additionally found that the NHB women with GDM had a 3- to 4-fold risk of HDP compared to those without GDM. We observed similar findings in this study and additionally demonstrated the association of glycemia outside the range of PDM/GDM with HDP. Though the pathophysiology of HDP is not well understood, there is evidence that insulin resistance, an underlying mechanism of GDM, plays a role in the development of HDP [32,33,34]. There is also an increased understanding that adverse in-utero cardiometabolic milieu can have a permanent impact on the body composition and vascular health of offspring [35].

NHB women are disproportionately affected by HDP [36,37] and its related mortality [38]. Contributing factors to this race-ethnic disparity in HDP risk include systemic factors such as structural racism, income inequality, disparities in healthcare access, and individual factors such as advanced maternal age and cardiovascular risk factors. Glycemic status outside the range of PDM/GDM may need to be highlighted in addition to these factors that contribute to HDP risk in non-Hispanic Black women. Additionally, advanced maternal age and CVD risk factors such as pre-existing hypertension and obesity are common underlying risk factors of PDM/GDM [39,40] and HDP [41]. Our findings of a high prevalence of key CVD risk factors such as obesity and pre-existing hypertension in non-PDM/GDM women with moderate glycemia further highlight the need for risk stratification and weight management in such women.

Currently, no international consensus exists on criteria for GDM diagnoses, and treatment modalities (lifestyle or pharmacological) vary. Debates on lowering the threshold for GDM remain inconclusive, with some experts concerned that it would lead to increased prevalence of GDM diagnoses [42,43]. The primary aim of screening for and diagnosing GDM is to identify women at risk of short- or long-term complications. Clinicians need to acknowledge that this group of women with seemingly normal glycemia outside the range of GDM/PDM may be at risk of APOs and future cardiovascular risk. Such women may benefit from dietary and exercise improvements as well as weight management to reduce their risk of developing APOs and subsequent cardiovascular disease.

The key strength of this study is the use of a large racially diverse cohort that allows for exploration of APOs across the glycemic spectrum and sub-stratification by race-ethnicity. Moreover, clinical outcomes were based on physician diagnoses in electronic medical records, which reduced the possibility of misclassification. There are, however, a few limitations. First, the BBC does not have data on hyperlipidemia, physical activity, and diet, which are vital to the development of GDM, so they could not be explored. Future studies should explore the impact of these on maternal glycemia and APOs. Furthermore, the cohort is predominantly NHB and Hispanic, so other race-ethnicities could not be explored separately due to the limited sample sizes. Future studies may explore maternal glycemic spectrum and APOs in other race-ethnicities. Lastly, this is a single-center study and our findings remain to be replicated in other independent populations.

## 5. Conclusions

In this predominantly NHB and Hispanic cohort, similar to women with GDM and PDM, moderate maternal glycemia without PDM/GDM was associated with higher odds of HBW and HDP more strongly among NHB women. Additionally, non-PDM/GDM women with moderate glycemia had a higher prevalence of pre-existing hypertension and obesity compared to women with the lowest tertile of glucose. If further confirmed, modifications of current guidelines for glucose screening and risk stratification of women may be warranted.

## Figures and Tables

**Table 1:** Comparison of Maternal and Birth Characteristics Between the Maternal Glycemic Subgroups.

Maternal and Birth Characteristics	Maternal Glycemic Subgroups during Pregnancy (Mutually Exclusive)					*p*-Value
	without PDM or GDM			GDM	PDM	
	T1 of 1 h Glucose (50–95 mg/dL) *n* = 1166	T2 of 1 h Glucose (96–116 mg/dL) *n* = 1151	T3 of 1 h Glucose (117–201 mg/dL) *n* = 913	*n* = 582	*n* = 307	
**Age, *n* (%)**						**<0.001**
<20	158 (13.6)	111 (9.6)	66 (7.2)	16 (2.8)	14 (4.5)	
20–34	875 (75.0)	869 (75.5)	639 (70.0)	393 (67.5)	201 (65.5)	
≥35	133 (11.4)	171 (14.9)	208 (22.8)	173 (29.7)	92 (30.0)	
**Race, *n* (%)**						**<0.001**
Non-Hispanic Black	499 (42.8)	435 (37.8)	307 (33.6)	163 (28.0)	111 (36.2)	
Hispanic	156 (13.4)	186 (16.2)	142 (15.6)	150 (25.8)	66 (21.5)	
Others *	511 (43.8)	530 (46.1)	464 (50.8)	269 (46.2)	130 (42.4)	
**Parity, *n* (%)**						**<0.001**
0	595 (51.0)	528 (45.9)	373 (40.9)	184 (31.6)	110 (35.8)	
≥1	571 (49.0)	623 (54.1)	540 (59.2)	398 (68.4)	197 (64.2)	
**Educational Status, *n* (%)**						**0.060**
Secondary or less	291 (25.0)	257 (22.3)	224 (24.5)	174 (29.9)	88 (28.7)	
GED/High school	402 (34.5)	409 (35.5)	307 (33.6)	183 (31.4)	94 (30.6)	
College and above	473 (40.5)	485 (42.2)	382 (41.9)	225 (38.7)	125 (40.7)	
**Pre-existing Hypertension, *n* (%)**						**<0.001**
No	1133 (97.2)	1091 (94.8)	856 (93.7)	526 (90.4)	234 (76.2)	
Yes	33 (2.8)	60 (5.2)	57 (6.3)	56 (9.6)	73 (23.8)	
**Smoking in pregnancy, *n* (%)**						**0.004**
No	994 (85.2)	1023 (88.9)	809 (88.6)	527 (90.6)	260 (84.7)	
Yes	172 (14.8)	128 (11.1)	104 (11.4)	55 (9.5)	47 (15.3)	
**Body Mass Index, *n* (%)**						**<0.001**
<25	665 (57.0)	556 (48.3)	413 (45.2)	159 (27.3)	72 (23.5)	
25–29.9	280 (24.0)	324 (28.2)	241 (26.4)	172 (29.6)	91 (29.6)	
≥30	155 (13.3)	208 (18.1)	209 (22.9)	212 (36.4)	121 (39.4)	
Missing	66 (5.7)	63 (5.5)	50 (5.5)	39 (6.7)	23 (7.5)	
**Sex of infant, *n* (%)**						**0.514**
Female	588 (50.4)	565 (49.1)	453 (49.6)	294 (50.5)	138 (45.0)	
Male	578 (49.6)	586 (50.9)	460 (50.4)	288 (49.5)	169 (55.0)	
**Gestational age(weeks), *n* (%)**						**<0.001**
<37	235 (20.2)	240 (20.8)	189 (20.7)	192 (33.0)	132 (43.0)	
≥37	931 (79.8)	911 (79.2)	724 (79.3)	390 (67.0)	175 (57.0)	
**Birthweight (g), *n* (%)**						**<0.001**
<4000	1120 (96.1)	1080 (93.8)	845 (92.6)	500 (85.9)	285 (92.8)	
≥4000	46 (4.9)	71 (6.2)	68 (7.4)	82 (14.1)	22 (7.2)	

T1—1 h glucose 50–95 mg/dL. T2—1 h glucose 96–116 mg/dL. T3—1 h glucose 117–201 mg/dL. *p*-value < 0.05—significant. * Others include non-Hispanic white, Asian. GED—General Educational Development. PDM—Pregestational DM. GDM—Gestational DM.

**Table 2:** Crude and Adjusted Odds Ratios with 95% Confidence Intervals for the Association of Maternal Glycemic Subgroups (with/without Pre-existing Hypertension) with Hypertensive Disorders of Pregnancy ^†^.

Maternal Glycemic Subgroups with/without Pre-Existing Hypertension	Crude		Adjusted *	
	OR [95%CI]	*p*-Value	OR [95%CI]	*p*-Value
T1 with no pre-existing hypertension	reference		reference	
T2 with no pre-existing hypertension	1.50 [1.14–1.97]	**0.003**	1.47 [1.11–1.93]	**0.007**
T3 with no pre-existing hypertension	1.44 [1.09–1.90]	**0.011**	1.37 [1.03–1.82]	**0.031**
T1 with pre-existing hypertension	8.83 [4.51–17.29]	**<0.001**	7.63 [3.81–15.30]	**<0.001**
T2 with pre-existing hypertension	7.18 [4.16–12.39]	**<0.001**	5.91 [3.36–10.40]	**<0.001**
T3 with pre-existing hypertension	7.59 [4.62–12.46]	**<0.001**	6.59 [3.89–11.17]	**<0.001**

^†^ Hypertensive disorders of Pregnancy—Gestational hypertension, Preeclampsia, Eclampsia, HELLP. T1—1 h glucose 50–95 mg/dL. T2—1 h glucose 96–116 mg/dL. T3—1 h glucose 117–201 mg/dL. * adjusted for maternal age, parity, race, educational status, smoking in index pregnancy, body mass index, and sex of infant. *p*-value < 0.05—significant.

**Table 3:** Crude and Adjusted Odds Ratios with 95%CI for the Association of Maternal Glycemic Subgroups with Hypertensive Disorders of Pregnancy among NHB, Hispanic, and Women of Other Race-ethnicity Groups.

Maternal Glycemic Subgroups	Crude		Adjusted *	
	OR [95%CI]	*p*-Value	OR [95%CI]	*p*-Value
**Non-Hispanic Black women (*n* = 1515)**				
T1	1.00 [Ref]		1.00 [Ref]	
T2	1.82 [1.22–2.72]	**0.003**	1.67 [1.13–2.51]	**0.013**
T3	1.87 [1.22–2.88]	**0.004**	1.68 [1.07–2.62]	**0.023**
Gestational DM	2.60 [1.60–4.21]	**<0.001**	2.09 [1.25–3.49]	**<0.001**
Pregestational DM	2.30 [1.31–4.04]	**<0.001**	1.84 [1.02–3.32]	**<0.001**
**Hispanic women (*n* = 700)**				
T1	1.00 [Ref]		1.00 [Ref]	
T2	0.53 [0.25–1.13]	0.102	0.54 [0.25–1.18]	0.120
T3	0.64 [0.29–1.41]	0.273	0.58 [0.26–1.29]	0.181
Gestational DM	1.84 [0.97–3.47]	0.061	1.70 [0.82–3.52]	0.151
Pregestational DM	2.66 [1.27–5.57]	**0.009**	2.40 [1.07–5.39]	**<0.001**
**Women of Other Race-ethnicities** **^†^ (*n* = 1904)**				
T1	1.00 [Ref]		1.00 [Ref]	
T2	1.74 [1.17–2.58]	**0.006**	1.63 [1.09–2.44]	**0.017**
T3	1.26 [0.82–1.93]	0.302	1.14 [0.73–1.77]	0.559
Gestational DM	3.19 [2.09–4.87]	**<0.001**	2.60 [1.68–4.05]	**<0.001**
Pregestational DM	3.13 [1.86–5.24]	**<0.001**	2.51 [1.47–4.30]	**<0.001**

^†^ Other race-ethnicities include non-Hispanic White and Asian. T1—1 h glucose 50–95 mg/dL. T2—1 h glucose 96–116 mg/dL. T3—1 h glucose 117–201 mg/dL. * adjusted for maternal age, parity, race, educational status, smoking in index pregnancy, body mass index, and sex of infant. *p*-value < 0.05—significant.

## Data Availability

Data will be available upon reasonable request.

## Supplementary Materials

The following supporting information can be downloaded at: https://www.mdpi.com/article/10.3390/jcdd9060179/s1, Table S1: Crude and Adjusted Odds Ratios with 95% Confidence Intervals for the Association of Maternal Glycemic Subgroups with Preterm Birth; Table S2: Sensitivity analysis—Crude and Adjusted Odds Ratios with 95% Confidence Intervals for the Association of Maternal Glycemic Subgroups with Spontaneous Preterm Birth; Table S3: Association of Maternal Glycemic Sub-groups with Birth Weight; Table S4:Crude and Adjusted Odds Ratios with 95% Confidence In-tervals for the Association of Maternal Glycemic Subgroups with Cesarean section; Table S5: Crude and Adjusted Odds Ratios with 95%CI for the Association of Maternal Glycemic Subgroups with Preterm Birth among NHB, Hispanic and Women of Other Race-ethnicities; Table S6: Crude and Adjusted β-coefficient and Standard Errors (SE) for the Association of Maternal Glycemic Subgroups with Birth Weight among NHB, Hispanic and Women of Other Race-ethnicities; Table S7: Crude and Adjusted Odds Ratios with 95%CI for the Association of Maternal Glycemic Sub-groups with High Birth Weight among NHB, Hispanic and Women of Other Race-Ethnicities; Table S8: Crude and Adjusted Odds Ratios with 95%CI for the Association of Maternal Glycemic Sub-groups with Cesarean Section among NHB, Hispanic and Women of Other Race-ethnicities.

## Abbreviations

APOs	adverse pregnancy outcomes
aOR	adjusted odds ratio
BMI	body mass index
CS	cesarean section
GDM	gestational diabetes
HBW	high birth weight
HDP	hypertensive disorders of pregnancy
NHB	non-Hispanic Black
OGTT	oral glucose tolerance test
PDM	pregestational diabetes
PTB	preterm birth
