# Supplementary material for: Maternal Glycemic Spectrum and Adverse Pregnancy and Perinatal Outcomes in a Multiracial US Cohort
Source: J Cardiovasc Dev Dis. 2022 Jun 4;9(6):179. doi: 10.3390/jcdd9060179 (PMC9224544; doi:10.3390/jcdd9060179)
Supplement: Supplementary file 1 [file jcdd-09-00179-s001.zip › jcdd-1724647-supplementary.pdf]

Supplementary Tables

Table S1: Crude and Adjusted Odds Ratios with 95% Confidence Intervals for the Association of Maternal Glycemic Subgroups with Preterm Birth

**Maternal Glycemic Subgroups**	**Crude** OR [95%CI]	p-value	**Adjusted*** OR [95%CI]	p-value
T1	reference		reference	
T2	1.04 [0.85-1.28]	0.678	1.06 [0.86-1.30]	0.583
T3	1.03 [0.83 -1.28]	0.759	1.04 [0.83 -1.29]	0.736
Gestational DM	1.95 [1.56-2.44]	**<0.001**	2.12 [1.66-2.70]	**<0.001**
Pregestational DM	2.99 [2.29-3.90]	**<0.001**	2.87 [2.16-3.80]	**<0.001**

Preterm birth - gestational age < 37 weeks

T1 – 1h glucose 50-95mg/dl

T2- 1h glucose 96-116mg/dl

T3- 1h glucose 117-201mg/dl

*adjusted for maternal age, parity, race, educational status, smoking in index pregnancy, body mass index, sex of infant, birth weight

p-value <0.05 -significant

Table S2: Sensitivity analysis - Crude and Adjusted Odds Ratios with 95% Confidence Intervals for the Association of Maternal Glycemic Subgroups with Spontaneous Preterm Birth

**Maternal glycemic subgroups**	**Crude** OR [95%CI}	p-value	**Adjusted** OR [95%CI}	p-value
T1	Ref		Ref	
T2	0.95[0.74-1.21]	0.67	0.98[0.76-1.25]	0.87
T3	1.07 [0.83-1.39]	0.59	1.14[0.87-1.48]	0.34
Gestational DM	1.87[1.43-2.44]	**<0.001**	2.25[1.68-3.00]	**<0.001**
Pregestational DM	2.15 [1.53-3.01]	**<0.001**	2.26[1.59-3.22]	**<0.001**

T1 – 1h glucose 50-95mg/dl

T2- 1h glucose 96-116mg/dl

T3- 1h glucose 117-201mg/dl

*adjusted for maternal age, parity, race, educational status, smoking in index pregnancy, body mass index, sex of infant, birth weight

p-value <0.05 -significant

Table S3: Association of Maternal Glycemic Subgroups with Birth Weight

Crude and Adjusted β-coefficient (β) and Standard Errors(SE) of the Association of Maternal Glycemic Subgroups with Birth Weight

**Maternal Glycemic Subgroups**	**Crude**			**Adjusted***		
	β	SE	p-value	β	SE	p-value
T1	reference	reference	reference	reference	reference	reference
T2	53.74	30.18	0.075	31.68	24.07	0.118
T3	166.43	32.09	**<0.001**	132.61	25.83	**<0.001**
Gestational DM	146.82	36.86	**<0.001**	192.89	30.70	**<0.001**
Pregestational DM	-82.33	46.59	0.077	76.87	38.25	**0.045**

Crude and Adjusted Odds Ratios with 95% Confidence Intervals for the Association of Maternal Glycemic Subgroups with High Birth Weight

**Maternal Glycemic Subgroups**	**Crude**		**Adjusted***	
	OR [95%CI]	p-value	OR [95%CI]	p-value
T1	reference		Reference	
T2	1.60 [1.09-2.34]	**0.015**	1.47 [1.01-2.19]	**0.044**
T3	1.96[1.33-2.88]	**0.001**	1.68 [1.13-2.50]	**0.010**
Gestational DM	3.99 [2.74-5.82]	**<0.001**	3.62 [2.42-5.41]	**<0.001**
Pregestational DM	1.88 [1.11 – 3.18]	**0.018**	1.89 [1.09-3.28]	**0.019**

High birth weight – birth weight ≥4000g

T1 – 1h glucose 50-95mg/dl

T2- 1h glucose 96-116mg/dl

T3- 1h glucose 117-201mg/dl

*adjusted for maternal age, parity, race, educational status, smoking in index pregnancy, body mass index, sex of infant and preterm birth

p-value <0.05 -significant

Table S4: Crude and Adjusted Odds Ratios with 95% Confidence Intervals for the Association of Maternal Glycemic Subgroups with Cesarean section

**Maternal Glycemic Subgroups**	**Crude**		**Adjusted***	
	OR [95%CI]	p-value	OR [95%CI]	p-value
T1	reference		reference	
T2	1.26 [1.06-1.50]	**0.010**	1.16 [0.97 -1.39]	0.106
T3	1.24 [1.03- 1.49]	**0.022**	1.06 [0.87 -1.28]	0.560
Gestational DM	2.30 [1.87 -2.82]	**<0.001**	1.63 [1.31-2.02]	<**0.001**
Pregestational DM	3.24 [2.50-4.20]	<**0.001**	2.20 [1.68-2.89]	<**0.001**

T1 – 1h glucose 50-95mg/dl

T2- 1h glucose 96-116mg/dl

T3- 1h glucose 117-201mg/dl

*adjusted for maternal age, parity, race, educational status, smoking in index pregnancy, body mass index, sex of infant, preterm birth

p-value <0.05 -significant

Table S5: Crude and Adjusted Odds Ratios with 95%CI for the Association of Maternal Glycemic Subgroups with Preterm Birth among NHB, Hispanic and Women of Other Race-ethnicities

Maternal Glycemic Subgroups	Crude		Adjusted*	
	OR [95% CI]	p-value	OR [95% CI]	p-value
**Non-Hispanic Black women**				
T1	1.00 [Ref]		1.00 [Ref]	
T2	1.08 [0.79-1.47]	0.648	1.10 [0.80-1.52]	0.550
T3	0.92 [0.65- 1.32]	0.657	0.93 [0.64-1.34]	0.692
Gestational DM	1.54 [1.03-2.30]	**0.036**	1.65 [1.07-2.55]	**0.024**
Pregestational DM	3.73 [2.43-5.74]	**<0.001**	3.66 [2.31-5.80]	**<0.001**
**Hispanic women**				
T1	1.00 [Ref]		1.00 [Ref]	
T2	0.89[0.53-1.52]	0.680	0.86 [0.51-1.49]	0.610
T3	0.76[0.42-1.36]	0.352	0.79[0.44-1.42]	0.430
Gestational DM	2.04 [1.22-3.39]	**0.006**	2.09 [1.19-3.68]	**0.010**
Pregestational DM	2.75 [1.48-5.11]	**0.001**	2.68[1.37-5.24]	**0.004**
**Women of other race-ethnicities†**				
T1	1.00 [Ref]		1.00 [Ref]	
T2	1.08[0.79-1.46]	0.627	1.10[0.81-1.51]	0.528
T3	1.23[0.90-1.68]	0.185	1.25[0.92-1.72]	0.170
Gestational DM	2.19[1.57-3.06]	**<0.001**	2.40[1.69-3.43]	**<0.001**
Pregestational DM	2.54[1.68-3.87]	**<0.001**	2.35[1.52-3.64]	**<0.001**

Preterm birth - gestational age < 37 weeks

T1 – 1h glucose 50-95mg/dl

T2- 1h glucose 96-116mg/dl

T3- 1h glucose 117-201mg/dl

*adjusted for maternal age, educational status, parity, smoking in index pregnancy, body mass index, sex of infant, birth weight

†Other race-ethnicities include non-Hispanic White and Asian

Table S6: Crude and Adjusted β-coefficient and Standard Errors (SE) for the Association of Maternal Glycemic Subgroups with Birth Weight among NHB, Hispanic and Women of Other Race-ethnicities

**Maternal Glycemic Subgroups**	**Crude**			**Adjusted***		
	β	SE	p-value	β	SE	p-value
**Non-Hispanic Black women**						
T1	Reference			Reference		
T2	38.44	49.17	0.434	29.53	37.67	0.433
T3	177.73	54.37	**0.001**	127.88	42.15	**0.002**
Gestational DM	140.58	67.62	**0.038**	172.47	53.60	**0.001**
Pregestational DM	-160.23	78.66	**0.042**	96.25	62.20	0.122
**Hispanic women**						
T1	Reference			Reference		
T2	77.03	75.50	0.308	54.49	63.89	0.394
T3	231.30	80.66	**0.004**	175.65	68.07	**0.010**
Gestational DM	203.70	79.53	**0.011**	218.53	72.56	**0.003**
Pregestational DM	43.12	102.12	0.673	114.01	90.12	0.206
**Women of Other Race-ethnicities†**						
T1	Reference			Reference		
T2	49.67	44.39	0.263	33.54	35.79	0.349
T3	126.17	45.92	**0.006**	126.88	37.32	**0.001**
Gestational DM	92.16	53.94	0.088	174.62	44.69	**<0.001**
Pregestational DM	-93.80	70.34	0.182	39.63	57.94	0.494

T1 – 1h glucose 50-95mg/dl

T2- 1h glucose 96-116mg/dl

T3- 1h glucose 117-201mg/dl

*adjusted for maternal age, educational status, parity, smoking in index pregnancy, body mass index, sex of infant, preterm birth

p-value <0.05 -significant

†Other race-ethnicities include non-Hispanic White and Asian

Table S7: **Crude and Adjusted Odds Ratios with 95%CI for the Association of Maternal Glycemic Subgroups with High Birth Weight among NHB, Hispanic and Women of Other Race-Ethnicities**

Maternal Glycemic Subgroups	Crude		Adjusted*	
	OR [95% CI]	p-value	OR [95% CI]	p-value
**Non-Hispanic Black women**				
T1	1.00 [Ref]		1.00 [Ref]	
T2	1.74[0.96-3.16]	0.070	1.67 [0.90-3.08]	0.101
T3	2.64 [1.45-4.79]	**0.001**	2.01 [1.08-3.74]	**0.028**
Gestational DM	4.79 [2.58-8.92]	**<0.001**	3.87 [1.97-7.59]	**<0.001**
Pregestational DM	1.70[0.70-4.15]	0.244	1.85 [0.72-4.76]	0.201
**Hispanic women**				
T1	1.00 [Ref]		1.00 [Ref]	
T2	1.72 [0.57-5.13]	0.334	1.47[0.48-4.52]	0.498
T3	2.54 [0.86-7.49]	0.092	2.31[0.77-7.00]	0.137
Gestational DM	4.92 [1.80-13.41]	**0.002**	3.97[1.36-11.74]	**0.012**
Pregestational DM	3.58 [1.09-11.74]	**0.035**	3.15[0.89-11.17]	0.075
**Women of other race-ethnicities†**				
T1	1.00 [Ref]		1.00 [Ref]	
T2	1.48[0.85-2.57]	0.168	1.37[0.78-2.41]	0.273
T3	1.43[0.80-2.53]	0.224	1.29[0.72-2.33]	0.394
Gestational DM	3.32[1.91-5.79]	**<0.001**	3.17[1.76-5.70]	**<0.001**
Pregestational DM	1.46[0.63-3.35]	0.375	1.40[0.59-3.37]	0.447

High birth weight - BW ≥4000g

T1 – 1h glucose 50-95mg/dl

T2- 1h glucose 96-116mg/dl

T3- 1h glucose 117-201mg/dl

*adjusted for maternal age, educational status, parity, smoking in index pregnancy, body mass index, sex of infant, preterm

p-value <0.05 -significant

†Other race-ethnicities include non-Hispanic White and Asian

Table S8: Crude and Adjusted Odds Ratios with 95%CI for the Association of Maternal Glycemic Subgroups with Cesarean Section among NHB, Hispanic and Women of Other Race-ethnicities.

Maternal Glycemic Subgroups	Crude		Adjusted*	
	OR [95% CI]	p-value	OR [95% CI]	p-value
**Non-Hispanic Black women**				
T1	1.00 [Ref]		1.00 [Ref]	
T2	1.17 [0.89-1.55]	0.254	1.09 [0.82 -1.44]	0.566
T3	1.19 [0.87-1.61]	0.273	1.04 [0.76 -1.43]	0.789
Gestational DM	2.00 [1.39-2.88]	**<0.001**	1.55 [1.06-2.27]	**0.024**
Pregestational DM	3.08 [2.02 – 4.70]	**<0.001**	2.12 [1.36-3.30]	**0.001**
**Hispanic women**				
T1	1.00 [Ref]		1.00 [Ref}	
T2	1.28 [0.81-2.03]	0.283	1.10[0.69-1.77]	0.685
T3	1.47 [0.91-2.38]	0.120	1.37[0.83-2.26]	0.225
Gestational DM	1.51 [0.94-2.43]	0.091	0.95 [0.55-1.62]	0.841
Pregestational DM	3.92 [2.14-7.18]	**<0.001**	2.68 [1.40-5.15]	**0.003**
**Women of other race-ethnicities**				
T1	1.00 [Ref]		1.00[Ref]	
T2	1.32[1.02-1.72]	**0.035**	1.24[0.95-1.63]	0.112
T3	1.22[0.93-1.60]	0.152	1.03[0.77-1.36]	0.841
Gestational DM	3.17[2.33-4.32]	**<0.001**	2.32[1.68-3.21]	**<0.001**
Pregestational DM	3.08[2.076-4.58]	**<0.001**	2.14[1.41-3.25]	**<0.001**

T1 – 1h glucose 50-95mg/dl

T2- 1h glucose 96-116mg/dl

T3- 1h glucose 117-201mg/dl

*adjusted for maternal age, educational status, parity, smoking in index pregnancy, body mass index, sex of infant, birth weight, preterm birth

p-value <0.05 -significant
